# A Versatile Multiple-Pass Raman System for Industrial Trace Gas Detection

**DOI:** 10.3390/s21217173

**Published:** 2021-10-28

**Authors:** Chunlei Shen, Chengwei Wen, Xin Huang, Xinggui Long

**Affiliations:** Institute of Nuclear Physics and Chemistry, China Academy of Engineering Physics, Mianyang 621900, China; shenchunlei@caep.cn (C.S.); chengweiw@caep.cn (C.W.); huangx2016@caep.cn (X.H.)

**Keywords:** industrial process control, multiple-pass Raman spectroscopy, multiple-point detection, multigas analysis

## Abstract

The fast and in-line multigas detection is critical for a variety of industrial applications. In the present work, we demonstrate the utility of multiple-pass-enhanced Raman spectroscopy as a unique tool for sensitive industrial multigas detection. Instead of using spherical mirrors, D-shaped mirrors are chosen as cavity mirrors in our design, and 26 total passes are achieved in a simple and compact multiple-pass optical system. Due to the large number of passes achieved inside the multiple-pass cavity, experiments with ambient air show that the noise equivalent detection limit (3σ) of 7.6 Pa (N_2_), 8.4 Pa (O_2_) and 2.8 Pa (H_2_O), which correspond to relative abundance by volume at 1 bar total pressure of 76 ppm, 84 ppm and 28 ppm, can be achieved in one second with a 1.5 W red laser. Moreover, this multiple-pass Raman system can be easily upgraded to a multiple-channel detection system, and a two-channel detection system is demonstrated and characterized. High utilization ratio of laser energy (defined as the ratio of laser energy at sampling point to the laser output energy) is realized in this design, and high sensitivity is achieved in every sampling position. Compared with single-point sampling system, the back-to-back experiments show that LODs of 8.0 Pa, 8.9 Pa and 3.0 Pa can be achieved for N_2_, O_2_ and H_2_O in one second. Methods to further improve the system performance are also briefly discussed, and the analysis shows that similar or even better sensitivity can be achieved in both sampling positions for practical industrial applications.

## 1. Introduction

Optical spectroscopy is one of the most important techniques for multigas analysis since optical spectroscopy techniques are nondestructive and noncontact and allow for in situ monitoring. Traditional multigas analysis techniques include gas chromatography (GC), mass spectroscopy (MS) and infrared (IR) absorption spectroscopy. The analysis speed is relatively slow for GC. Though MS is very sensitive, the instrument is rather expensive, and a lot of calibration efforts are needed for quantitative analysis. Infrared absorption-based technologies, such as tunable diode laser spectroscopy (TDLAS) [1], photoacoustic spectroscopy (PAS) [2] or cavity ring-down spectroscopy (CRDS) [3], are most commonly used since these techniques provide extraordinary sensitivities and selectivity. However, important diatomic homonuclear molecules (e.g., H_2_, N_2_) are challenging to detect with infrared-based techniques. Besides, several laser sources with different wavelengths are required for multigas detection.

Raman spectroscopy, on the other hand, allows for simultaneous identification of almost all gases (e.g., H_2_, CO_2_ and hydrocarbons, except for monatomic gases) with a single laser source. Due to different selection rules, Raman spectroscopy can also be used to target important diatomic homonuclear molecules. These molecules are particularly relevant for many fields, such as power transformer diagnosis [4], medical gas sensing [5,6], biogas analysis [7,8] and process control in nuclear reactors [9,10]. The main disadvantage of Raman spectroscopy is the low Raman signal intensity due to small scattering cross section of gas molecules and low molecular density in the gas phase. Thus, for Raman spectroscopy to achieve widespread use in scientific and industrial applications, the Raman signal of gas molecules must be enhanced substantially. In the past few years, various Raman systems have been designed and implemented, aiming at lowering limit of detection (LOD) of gas molecules. Examples of such systems are cavity-enhanced Raman spectroscopy (CERS) [11,12,13,14,15], fiber-enhanced Raman spectroscopy (FERS) [16,17,18,19,20,21], Purcell-enhanced Raman spectroscopy [22,23] and multiple-pass-enhanced Raman spectroscopy [24,25,26,27,28,29,30,31].

Among various techniques, the multiple-pass optical system is the easiest way to realize high sensitivity, though usually the gain factor is limited compared to other techniques. In a multiple-pass system, the optical system design is aimed at increasing the laser energy in a small collection volume, and the multiple reflections of light are ultimately responsible for the resulted high sensitivity. Petrov described a near-concentric multiple-pass Raman system based on 90-degree geometry Raman light collection. With 5 W laser output energy, LODs close to 50 ppm can be achieved in 30 s for main components of ambient air [26]. Recently, instead of using side detection geometry, Velez et al. employed a collinear detection geometry for their near-concentric multiple-pass cavity, and 34 ppm was achieved for CO2 in 5 s [27]. We have recently introduced a variant of multiple-pass Raman spectroscopy with enhanced sensitivity and stability for industrial long-term monitoring applications [29,30,31]. We take advantage of the large collection area of fiber bundles, which relaxes the laser beam overlap requirements inside a multiple-pass cell. The use of fiber bundle with large area also greatly improves the long-term stability and practicability of an industrial Raman system. With a closed gas chamber, this system is ideal for sensitive in-line monitoring of radioactive or corrosive gas species, as well as other nonhazardous gas samples.

Conventional multiple-pass optical systems for Raman detection usually adopt either (near) concentric or confocal cavity designs. As a result, spherical mirrors are used as cavity mirrors. Usually, the alignment is very tedious in those systems, and cavity mechanical stability is critical. In this contribution, we improve on the multiple-pass optical system developed previously. A highly sensitive and versatile multiple-pass Raman system has been established, mainly aiming for multiple point detection of trace nonhazardous gas samples. Instead of using spherical mirrors, D-shaped flat mirrors are chosen as cavity mirrors in our design, and 26 total passes are achieved inside the compact multiple-pass cavity. Alignment of this multiple-pass system is extremely simple and straightforward. With help of these important improvements, noise equivalent detection limits (3σ) of 7.6 Pa (N_2_), 8.4 Pa (O_2_) and 2.8 Pa (H_2_O) are achieved in 1 s integration time with a 1.5 W red laser. This multiple-pass Raman system can be easily upgraded to a multiple-channel detection system, and a two-channel detection system is demonstrated and characterized. High utilization ratio of laser energy (defined as the ratio of laser energy at sampling point to the laser output energy) is realized in this design. As a result, high sensitivity is achieved in both sampling positions. Compared with the single-channel system, the back-to-back experiments show that LODs of 8.0 Pa, 8.9 Pa and 3.0 Pa can be achieved for N_2_, O_2_ and H_2_O. The results obtained with this multiple-pass Raman setup are very promising, and a variety of industrial applications can benefit from the current design.

## 2. Materials and Methods

**Figure 1:**
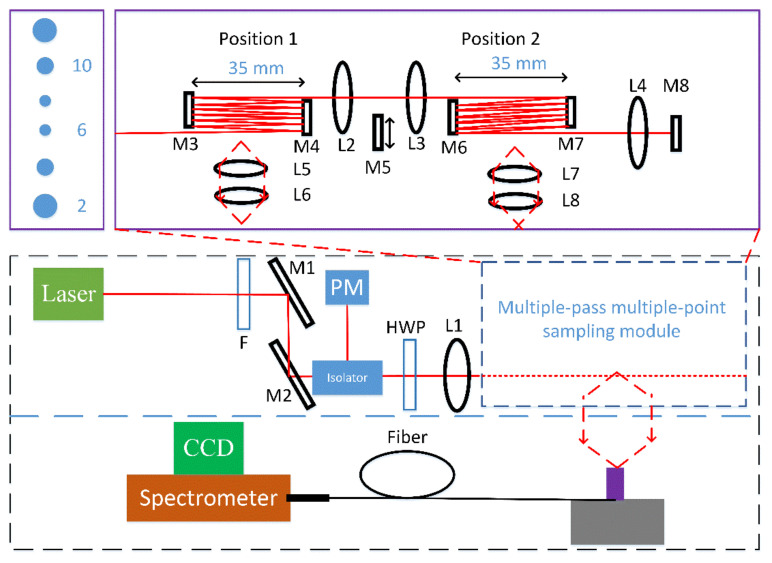
Scheme of the experimental setup. M, Mirrors; L, lenses; F, Filter; PM, power meter; HWP, half-wave plate.

The newly designed multiple-pass Raman system is shown schematically in Figure 1. The laser head (Laser Quantum OPUS660) is stabilized by a water cooler, which maintains the base plate temperature at 24 degrees Celsius. The OPUS660, in fact, was first chosen for hydrogen isotopologues monitoring applications in our previous systems [29,30,31]. We use 660 nm instead of a shorter wavelength (e.g., 532 nm) because, in our previous design, the gas chamber was located between the cavity mirrors, and thus, fluorescence generated from optical windows reduced the signal-to-noise ratio. For current system with a different gas chamber design, 532 nm or even shorter wavelength can also be used. A band-pass filter (Semrock, FF01-661/11) is used to remove any unwanted laser lines. The laser output beam is then guided by two highly reflective mirrors (M1 and M2) to pass an optical isolator. The dielectric coatings of mirror used in this experiment usually have approximately 99.5% reflectivity at the laser wavelength. After that, a half-wave plate is inserted to tune the polarization of the excitation beam to maximize gas Raman signal for 90-degree collection geometry. The beam is finally focused by a 300 mm focus lens (L1) into a multiple-pass optical system and reflected multiple times inside the multiple-pass cavity to increase the signal strength.

To enhance the Raman signals of nonhazardous gas species in the collection volume, a new multiple-pass scheme is designed. The multiple-pass cell used in our experiments mainly consists of two high-reflection D-shaped mirrors of 25 mm diameter (M3 and M4), and the alignment of this multiple-pass optical system is greatly simplified by not using spherical mirrors. Those D-shaped mirrors offer an advantage over traditional mirrors since they facilitate the separation of closely spaced beams. The cavity length (distance between M3 and M4) is about 35 mm and is greatly reduced compared with conventional (near) concentric systems and our previous designs. The distance between M3 and the focusing lens (L1) is approximately 10 cm. The exact distance between optical components is not that important in current design. Alignment of this multiple-pass system is extremely simple, and usually a couple of minutes are enough to complete the construction of the multiple-pass cavity. In the forward path, the incoming beam is first incident on mirror M4. After reflection from this mirror, the beam is incident on the edge of mirror M3. The laser beam is then reflected multiple times between M3 and M4 before it leaves the multiple-pass cell defined by M3 and M4. Six laser spots are clearly seen on both mirrors, though the diameters of laser spots are slightly different (spot pattern on M3 is show schematically in Figure 1, top left). The lateral separation of excitation beams in the collection volume is about 8 mm. This excitation geometry gives a total forward pass of 13 (single pass configuration). Using beam diameter of about 1.1 mm and lens focus of 300 mm, the beam diameter at the focus is 228 um and approximately 700 um for the first and last passes. The beam diameter for other passes will be in between. The out-going beam is then collimated by a second lens with focus of 300 mm and is finally reflected back by mirror M5 to double the number of passes (double-pass configuration). The back-going beam is finally deflected out of the beam path by an isolator to avoid any back-reflection of laser beam into the laser head. Thus, 26 total passes are achieved in this multiple-pass system. During alignment, the laser beams should not clip the sharp edge of the D-shaped mirror in order to minimize formation of interference fringes. Compared with conventional two-concave mirror designs, current multiple-pass system is characterized by its simplicity of alignment and compactness, as well as its adjustment stability.

Another advantage of current design is that the multiple-pass optical system described above can be easily upgraded to a multiple-channel detection system. For example, for a two-channel detection system, the mirror M5 is removed, and lens L3 with a focus of 300 mm is used to refocus the collimated laser beam into another multiple-pass cell defined by mirrors M6 and M7. The two sampling regions where the Raman signal can be collected are named positions 1 and 2, as also indicated in Figure 1. The incoming beam is then reflected back and forth inside the multiple-pass cavity to give exactly 13 total passes. The out-going laser beam is then collimated by lens L4 with a focus of 300 mm. Finally, mirror M8 is used to double the number of passes in both multiple-pass cavities. Thus, 26 total passes are achieved in both sampling positions.

The gas Raman signals are collected by a pair of achromatic lenses (L5 and L6, with focal lengths of 80 mm and 50 mm diameter) at a right angle to the excitation beam and 1:1 imaged onto a fiber bundle comprising 60 multimode fibers (N.A. = 0.22, core diameter 100 um) arranged in a rectangular-to-slit configuration. For the two-channel detection system, another pair of achromatic lenses (L7 and L8, with focal lengths of 80 mm and 50 mm diameter) is installed to collect the gas Raman signals at position 2. The collection end of the fiber has a dimension of approximately 0.7 × 1.5 mm to match the beam diameter in the collection volume. The output end is arranged as a curved slit with approximately 7 mm height. This allows the full binning of vertical pixels without sacrifice resolution. Typical resolution of our system is about 25 cm^−1^. For applications where higher resolution is required, either a grating of higher density can be used or multimode fiber with smaller core diameter can be selected. The scattered light is then coupled into a Kaiser Optics f/1.8i high throughout spectrograph. This system contains no moving parts to ensure long system stability and is suitable for industrial applications. The Raman spectra were finally recorded by a CCD detector (PIXIS 400BRX) operating at −74 degrees Celsius.

## 3. Results

### 3.1. Performance of Current Multiple-Pass Raman System

Compared with our previous multiple-pass setups, the multiple-pass cavity length is greatly reduced in the new design, and it is impossible to insert a closed gas chamber between cavity mirrors. The current setup can be directly used to monitor gas species in an atmosphere environment. For example, multiple consecutive breaths from different people can be exhaled into the sampling positions using Teflon tubes [24,25]. For applications where a closed gas chamber is needed, a slight modification of current configuration can be adopted, and the multiple-pass cavities (M3, M4 and M6, M7) can be placed inside two closed gas chambers [4,11,26]. For example, the system can be applied to power transformer diagnosis and logging gas detection, and the gas samples can be sent to the (multiple) closed gas chambers through a valve system. Both configurations have the advantage that no fluorescence background is generated in the excitation region. To demonstrate performance and sensitivity of this multiple-pass Raman system, spectra of ambient air were recorded without a gas cell.

**Figure 2:**
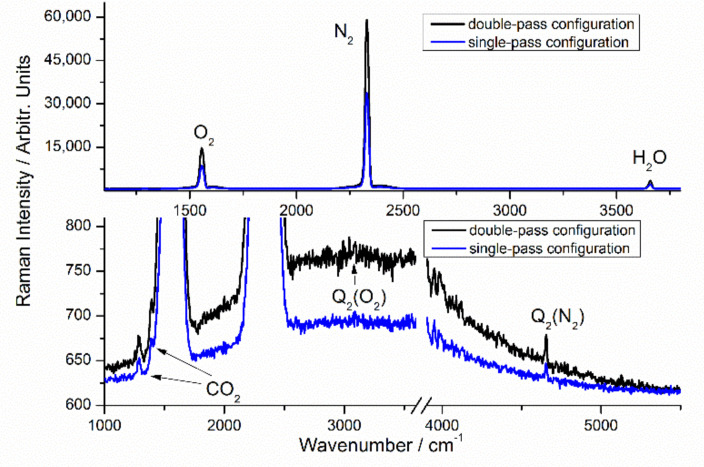
Raw spectra of ambient air with 1 s integration time. Top: Spectral overview. Bottom: Low-intensity parts of spectra.

**Figure 3:**
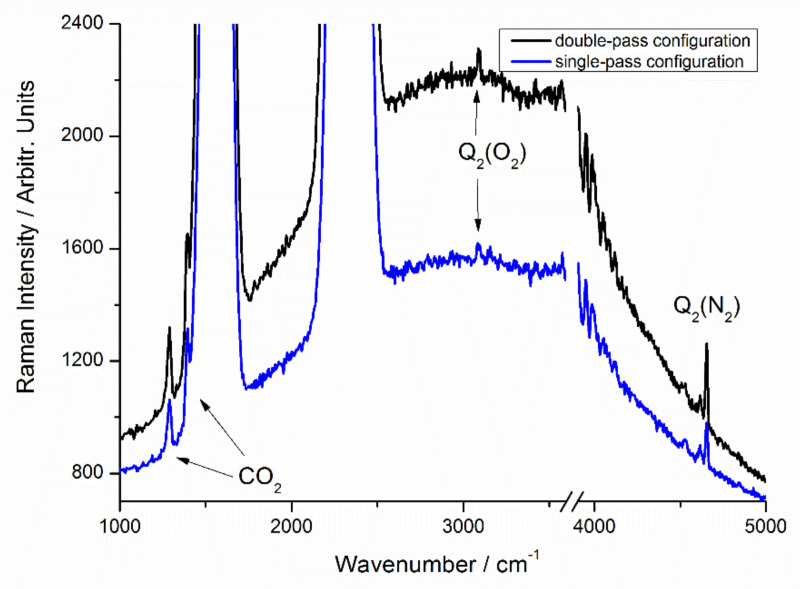
Low-intensity parts of raw spectra with 10 s integration time. Note that with 10 s integration time, the Q-branch peaks (not shown) of O_2_ and N_2_ are saturated in the detector.

For the double pass configuration, the spectrum of ambient air is shown in Figure 2 and Figure 3. For these experiments, the laser output power was set to 1.5 W. The spectrum of ambient air is dominated by the spectral features of oxygen (O_2_) at 735.5 nm (1555.2 cm^−1^), nitrogen (N_2_) at 779.9 nm (2329.9 cm^−1^) and water molecules (H_2_O) at 869.9 nm (3656.2 cm^−1^). Besides major components, the spectral features of Q_2_ (N_2_) branch at 952.8 nm (4656.0 cm^−1^) and CO_2_ at 721.1 nm (1283.6 cm^−1^) and 726.6 nm (1388.7 cm^−1^) can also be unequivocally assigned with 1 s integration time (Figure 2, bottom), though the contrast of the higher wavenumber peak of the CO_2_ Fermi resonance pair has been degraded by the Q_1_ (O_2_) branch. The signature of Q_2_ (O_2_) branch at 828.9 nm (3087.6 cm^−1^) is almost noise-limited with 1 s integration time but is detectable with 10 s integration time, as shown in Figure 3. The above results clearly demonstrate the high sensitivity achieved in this Raman setup, considering that the intensity of Q_2_ (O_2_) branch is approximately 3000 times less than Q_1_ (O_2_) branch. The LODs are estimated using procedures described in detail in a previous publication and is associated with a signal-to-noise ratio (SNR) of 3 [29]. In the current work, noise is calculated using flat regions between 5400 to 5700 cm^−1^. For N_2_ and O_2_ (assuming 78% and 21% composition in the air) with 1 s integration time, the noise (σ) is extracted as 1.96. The estimated noise equivalent detection limits (3σ) are 7.6 Pa and 8.4 Pa for N_2_ and O_2_, respectively, which corresponds to relative abundance by volume at 1 bar total pressure of 76 ppm and 84 ppm. From the humidity data logger reading of 55% RH (corresponding to approximately 1265 Pa of water vapor in the air), a LOD of 2.8 Pa is deduced for H_2_O, which corresponds to relative abundance by volume at 1 bar total pressure of 28 ppm. Recently, Godot et al. have introduced a commercial Raman analyzer for process control in a tritium facility, and the LOD was reported as 200 Pa for hydrogen isotopologues, with an acquisition time of 120 s [32]. Based on our previous investigations [29,30,31], the LOD of the current setup for hydrogen isotopologues can be safely estimated to be lower than that of H_2_O. Thus, the current system is preferred in low-pressure gas applications. In recent year, FERS has shown excellent sensitivity for multigas analysis. Hanf et al. showed that LODs of 9 ppm and 8 ppm could be achieved for N_2_ and O_2_ [33]. For H_2_S, LOD as low as 33 ppm was also demonstrated [20]. Besides, small samples volumes are needed in this technique, which is critical for certain applications where amount of gas sample is limited. However, the small core diameter hampers the quick exchange of gas samples, and this technique is not suitable for in-line low-pressure gas detection and monitoring.

We have also tested a slightly different version of the single-pass configuration, where the isolator and half-wave plate are removed. A power meter is also inserted between L3 and L2 to safely collect the laser light and monitor laser performance. First, this configuration gives a simpler Raman system and should improve system robustness, as the number of optical components is reduced. Besides, full laser energy can be used for excitation, as approximately 15% laser energy is lost after passing the isolator and half-wave plate. The recorded spectra of ambient air are also shown in Figure 2 and Figure 3 with different integration times. Compared with double-pass configuration, the LODs for gas molecules have been increased by a factor of about 1.6. For certain applications, the achieved sensitivity is still acceptable, and single-pass configuration provides a simpler and lower-cost solution.

### 3.2. Characterization of the Two-Channel Detection System

With the development of science and technology, industrial monitoring applications also have even higher requirements for gas sensor systems. Besides high sensitivity and long-term stability, some applications require that the Raman system can be operated in an economical manner. The multiple-channel detection scheme greatly reduces the examination costs of a monitoring system and thus has drawn extensive attention in industrial multigas analysis applications. In real industrial gas detection applications, different gas samples can be transported to different detection positions (e.g., different gas chambers) through valve–pipeline systems. Thus, simultaneous composition monitoring at different sampling positions are realized using the same laser source and spectrometer. To demonstrate the sensitivity of this newly designed two-channel detection system, spectra of ambient air were recorded back-to-back at positions 1 and 2. The detailed experimental procedure is as follows: The spectra of lab air were recorded first in position 1. After data collection in position 1, the fiber bundle was removed and reinstalled and optimized in position 2. The spectra of lab air were then recorded in position 2. It should be noted that for these experiments the same fiber bundle is used, though in practical situations, signals can be collected simultaneously at multiple sampling positions through a branched fiber bundle.

**Figure 4:**
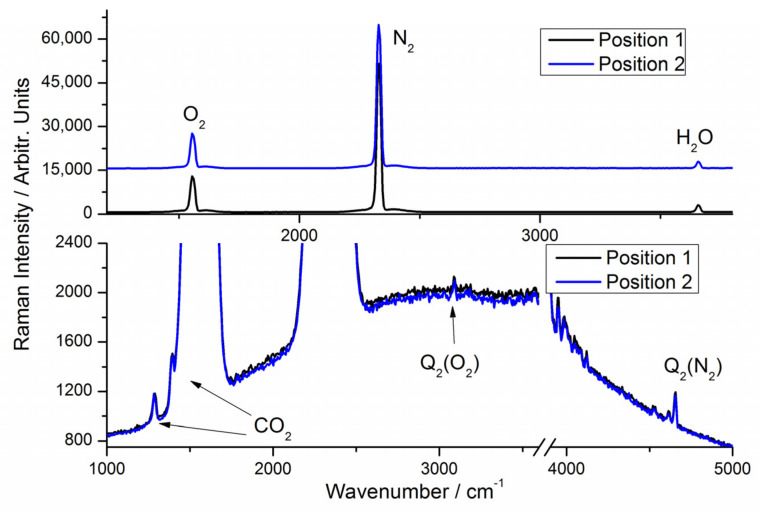
Raw spectra of ambient air at sampling positions 1 and 2. Top: Spectral overview with 1 s integration time. Traces are offset by 15,000 units. Bottom: Low-intensity parts of spectra with 10 s integration.

For the two-channel detection system, the spectra of ambient air recorded with laser output set to be 1.5 W is shown in Figure 4. The spectra of ambient air (Figure 4, top) recorded in positions 1 and 2 are nearly indistinguishable by visual inspection. The small difference in signal strength is due to slightly different alignments. With 10 s integration time, the peaks of Q_2_ (N_2_) and CO_2_ are readily identified, and the peak of Q_2_ (O_2_) is also distinguishable (Figure 4, bottom). Thus, similar high-sensitivity is also achieved in a two-channel detection system. At position 1 with 1 s integration time, experiments with ambient air show that the noise equivalent detection limit (3σ) of 8.0 Pa (N_2_), 8.9 Pa (O_2_) and 3.0 Pa (H_2_O) can be achieved, which corresponds to relative abundance by volume at 1 bar total pressure of 80 ppm, 89 ppm and 30 ppm. The LODs calculated at position 2 are almost identical to values obtained with position 1. The estimated LODs are slightly higher than the above (double-pass configuration) single-channel detection system, which is reasonable since the laser energy loss is higher in a two-channel detection system.

The above results clearly demonstrate sensitivity and capability of this Raman setup for multigas analysis. Due to similar design criteria, the long-term stability should also be comparable to our previous setups. Thus, this newly designed Raman system is especially suited for trace gas analysis in a number of industrial applications. Besides, the signal enhancement provided with current multiple-pass system can be further improved. Various approaches to improve the system performance have already been discussed thoroughly in a previous contribution, and we are not going to discuss them in detail [29,31]. For example, back reflection mirrors can be installed to double the collection solid angle; the total number of achievable passes can also be increased (e.g., 30 total passes) by adjusting the relative position (angle and distance) of the two cavity mirrors.

## 4. Conclusions

Sensitive multigas composition analysis and monitoring are needed for a variety of industrial applications. Usually, a gas analyzer that measures gas samples in a wide range of concentration and in real-time is required for process control applications. To this end, a highly sensitive, versatile and economical multiple-pass Raman system has been established and characterized, mainly aiming for multiple-point detection of trace nonhazardous gas samples. Instead of using spherical mirrors, D-shaped mirrors are chosen as cavity mirrors in this design, and 26 total passes are achieved inside a compact multiple-pass cavity. With 1.5 W of 660 nm excitation laser and 1 s integration time, cavity enhancement achieves LODs of 7.6 Pa (N_2_), 8.4 Pa (O_2_) and 2.8 Pa (H_2_O).

Moreover, this multiple-pass Raman system can be easily upgraded to a multiple-channel detection system, and a two-channel detection system is demonstrated and characterized. Compared with single-channel detection system, the noise equivalent detection limits at the two positions are almost identical and have been estimated to be 8.0 Pa (N_2_), 8.9 Pa (O_2_) and 3.0 Pa (H_2_O). The slightly higher LODs obtained in the two-channel detection system are mainly due to reflection loss at the mirrors and lenses, and the achieved LODs can be improved by using customized coatings. Other methods to improve the system performance are also briefly discussed, and the analysis indicates that similar or even better sensitivity can be achieved for every sampling position in a practical two-channel detection system. The results obtained with this multiple-pass Raman setup are very promising. The sensitivity can be further improved by using lasers with higher power since the LODs almost scale linearly with excitation laser power. Additionally, the Raman signal can be significantly increased by using lasers with shorter wavelengths. Thus, limits of detection in low ppm range for common gas samples are possible with exposure times of seconds.

## Data Availability

The data that support the findings of this study are available from the corresponding author upon reasonable request.
